# Effects of body mass index on mandibular bone architecture: a retrospective fractal dimension and radiomorphometric analysis

**DOI:** 10.1007/s00784-026-06833-8

**Published:** 2026-04-27

**Authors:** Nebiha Gozde Ispir, Gulsun Akay, Kahraman Gungor

**Affiliations:** Department of Oral and Dentomaxillofacial Radiology, Gazi University, Bişkek Street,1.Sk. No:4, Ankara, 06490 Turkey

**Keywords:** BMI, Fractal dimension, Mandibular bone, Radiomorphometric index, Obesity

## Abstract

**Objective:**

This retrospective study aimed to evaluate mandibular bone mineral density and bone trabecular structure using fractal dimension (FD) analysis and radiomorphometric indexes on dental panoramic radiographs, and to determine whether the results differed between groups with varying body mass index (BMI) levels.

**Materials and methods:**

Panoramic radiographs of 127 individuals aged 18–45 (84 females and 43 males, with a mean age of 30.37 ± 8.4 years) were retrospectively selected from clinical records. Individuals were divided into four groups based on their BMI percentiles: normal, underweight, overweight, and obese. FD measurements, Mental Index (MI), and Panoramic Mandibular Index (PMI) were evaluated. Kruskal-Wallis Test, Pearson Chi-Square Test, and Wilcoxon Test were used in the comparison of parameters.

**Results:**

MI and PMI values differed significantly among BMI groups (*p* = 0.005 and *p* = 0.025, respectively). Specifically, obese individuals had higher MI and PMI values compared to those in the other BMI categories. A statistically significant difference was also observed in FD premolar-canine values ​​among BMI categories (*p* < 0.001). However, no significant differences were found for FD premolar-molar, FD angulus, and FD condyle values ​​across BMI groups (*p* > 0.05).

**Conclusions:**

Mandibular cortex thickness (MI) values were thicker in obese and overweight adults, and PMI values were higher in obese individuals compared to lean individuals. However, only FD values measured in the premolar-canine region were observed to be lower in lean individuals compared to the other groups.

**Clinical relevance:**

The study findings demonstrate that BMI values ​​significantly affect mandibular cortical bone structure. Structural differences in cortical and trabecular bone may impact surgical procedures such as implant planning. Therefore, mandibular bone structure should be assessed preoperatively in cases of obesity and underweight to optimize primary stability and long-term success.

## Introduction

Obesity is defined by the World Health Organization (WHO) as a chronic and complex disease and remains a global public health problem with increasing prevalence across all age groups [1]. Body Mass Index (BMI) is a widely used measure to assess one’s degree of obesity in a straightforward manner [2].

The bone remodeling process is a dynamic, lifelong process that is influenced by various factors, including nutritional status, hormones, and blood calcium concentration [3]. However, the specific impact of obesity on bone health remains a subject of ongoing debate in the literature [4]. Some researchers argue that obesity may have negative effects on bone metabolism [5, 6], while others suggest that it may have a potentially protective role [7–9]. Obesity can affect bone tissue due to both mechanical and hormonal factors [10]. It has been reported that increased bone mineral density is caused by both mechanical effects and higher levels of estrogen found in adipose tissue, resulting from an increase in body weight [4]. However, inflammatory processes, including the release of cytokines by adipose tissue, can lead to bone destruction [11]. On the other hand, low BMI is a well-known risk factor for decreased mineral density and fragility fractures, highlighting the sensitivity of skeletal tissues to extreme weight changes [2].

A comprehensive assessment of mandibular bone quality and density is crucial before dental procedures and for monitoring overall systemic health. Changes in the jawbone can serve as an early indicator of systemic osteoporosis [12]. Bone quality is a collective term encompassing the mechanical properties of bone, its microarchitecture (cortical thickness and trabecular network distribution), the degree of matrix mineralization, and its capacity for bone remodeling [13]. Radiomorphometric mandibular indices, such as the Mental Index (MI) and Panoramic Mandibular Index (PMI), can be used to define osteoporosis and determine bone quality and characteristics, including the degree of mineralization of the bone matrix, cortical bone thickness, and structure [14]. MI and PMI are widely used in research to assess mandibular morphological changes and bone quality [15–17]. However, the literature reported that density measurement alone is insufficient for evaluating bone quality and that trabecular microarchitecture should also be examined [15]. Trabecular bone exhibits higher metabolic activity than cortical bone; its structural architecture is shaped by variations in thickness, porosity, and anisotropy [18, 19]. Therefore, assessment of trabecular microarchitecture in addition to cortical measurements is essential for a comprehensive evaluation of mandibular bone quality [15]. Fractal analysis (FA) quantitatively assesses the complexity and irregularity of trabecular bone structure (i.e., the bone meshwork) using the fractal dimension (FD), allowing for an objective evaluation of trabecular architecture [20]. Recent studies have employed FD analysis to evaluate the impact of various clinical conditions and diseases on the mandibular bone structure, such as TMJ degenerative changes [19, 21], cleft lip and palate patients [18], following dental implant procedures [22], and functional orthopedic treatments [23], alongside systemic diseases, including Behçet’s Disease [12], diabetes [16], and thyroid disorders and diabetes mellitus [24].

The mandibular bone is continuously subjected to complex biomechanical loading during mastication, speech, and parafunctional activities [25]. Differences in the density and quality of the mandibular bone provide critical data regarding the primary stability and long-term success of implant treatment [22]. Treatment duration and rate of tooth movement in orthodontic treatment planning [26], in the selection of osteotomy techniques, and in predicting complications such as unfavorable splits (also known as “bad splits”) in orthognathic surgery [27]. It has been reported that dense trabeculation in the mandibular bone is associated with higher bone mineral density in the skeleton, and high levels of obesity generally increase bone mineral density [3]. However, the effects of obesity on mandibular bone are still not fully understood [28]. In the literature, studies examining the relationship between obesity and mandibular bone structure are limited [10, 28]. Temur et al. [28] investigated potential changes in mandibular trabeculation and cortical bone in obese children and adolescents using FD analysis and radiomorphometric indices, while Yasa et al. [10] evaluated PMI and MI in adolescent patients with different BMI. To the best of our knowledge, there are no studies evaluating the relationship between BMI and mandibular microarchitecture using radiomorphometric mandibular indices and FD analysis together in adults. This study is the first to evaluate the relationship between BMI and mandibular bone density and quality using FD analyses and the radiomorphometric mandibular indices. The aim of this study was to evaluate the effect of BMI on mandibular bone quality by analyzing FD analyses and radiomorphometric indices (PMI and MI) on dental panoramic radiographs. The null hypothesis of our study is that there is no statistically significant difference between BMI categories and mandibular bone structure.

## Materials and methods

### Study groups

Before starting the study, ethics committee approval was obtained from the Gazi University Ethics Committee. (Research No:2022 − 275). All procedures were in accordance with the Helsinki Declaration. This study is a retrospective, single-center investigation. Patients aged 18 and over who applied to the Oral and Maxillofacial Radiology department for various dental reasons and underwent panoramic radiography within the specified parameters were included in the study. Inclusion criteria for the study were: individuals who did not have any systemic disease that would affect bone metabolism, no history of trauma, and no cyst/tumor-like lesions affecting the jaws. The power analysis of the study was conducted using G*Power 3.1 software, with an alpha level of 0.05 and a power value of 0.95% (1-β err prob), based on the study by Özden and Çiçek [18]. The minimum sample size was calculated to be 116 in total (29 per group), with a non-centrality parameter λ = 17.9200000 and a critical F-value of 2.6886915.

The height and weight of individuals who volunteered for the study were recorded, and BMI percentiles were calculated. BMI data were obtained by dividing the body weight in kilograms by the square of the height in meters. Since the eligibility criteria required participants to be 18 years of age or older, adult BMI measurements were used and categorized using international adult standards [29]. BMI is a widely used measurement to examine the weight status of the average population, as it is an accessible and cost-effective tool that requires only height and weight measurements to define individuals as underweight, normal weight, overweight, and obese [29]. In our study, we divided the sample group into four different groups according to the BMI percentile: Underweight group: BMI < 18.5; Normal weight group: BMI = 18.5 to 24.9; Overweight group: BMI = 25.0 to 29.9; Obese: BMI > 29.925.

All panoramic radiographs were performed using the same orthopantomography device (Sirona ORTHOPHOS XG, Sirona, Bensheim, Germany), with exposure parameters of 80 kVp, 8 mA, and a 14-second exposure time, and a 1.2 magnification factor. ImageJ v. 1.52 software program (National Institutes of Health, Bethesda, MD), which can be downloaded free of charge from https://imagej.nih.gov/ij/download.html, was used for fractal dimension analysis on panoramic radiography images. Panoramic radiography images were converted into tagged image file formats (TIFFs) due to their high resolution.

### Fractal dimension (FD) measurements

**Fig. 1:**
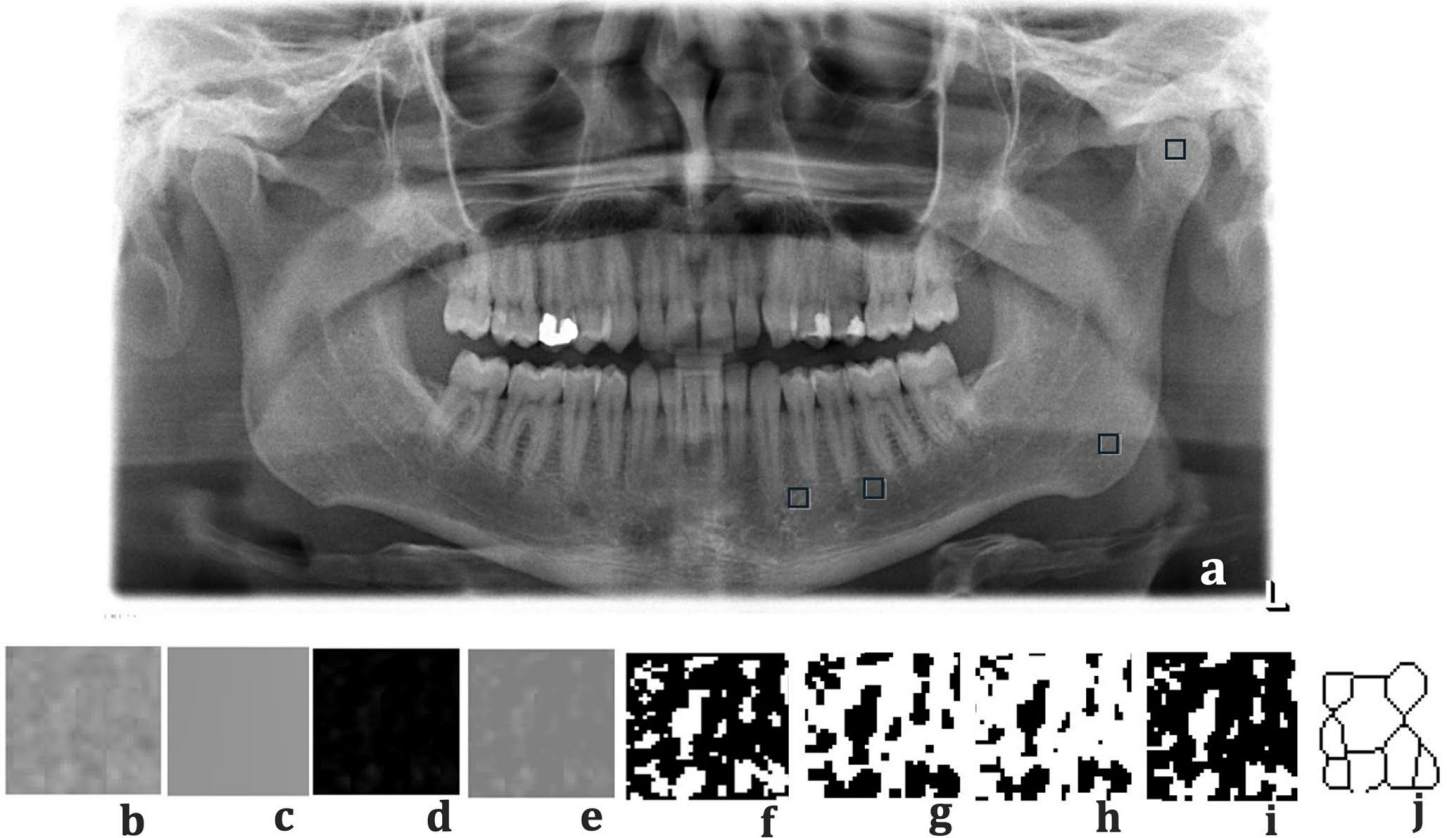
Shows the steps of fractal dimension analysis. Four 40×40-pixel square regions of interest (ROI) were selected (**a**), ROI was cropped and duplicated (**b**), and the “Gaussian Blur” filter (**c**). The blurred images were subtracted from the original image (**d**). The added image (**e**), the Binarised images (**f**), and the Eroded image (**g**). The Dilated image (**h**), the Inverted image (**i**), and the Skeletonized image (**j**)

FA was performed according to the box-counting method described by White-Rudolph [18]. For FD, four 40 × 40-pixel square regions of interest (ROI) were selected on the image (Fig. 1a). Four different ROIs were selected because they represent anatomically and functionally distinct mandibular regions and are frequently used in FD analysis studies [14, 16, 24]. These regions included the condyle, the mandibular angle, and two trabecular areas located superior to the mandibular canal: one distal to the second premolar (posterior to the mental foramen) and the other mesial to the first premolar (anterior to the mental foramen). ROI area did not include any teeth, cortical bone, or mandibular canal. Once selected, each ROI was cropped and duplicated (Fig. 1b). The “Gaussian Blur” filter (σ, 35 pixels) was applied to blur bright areas caused by changes in soft tissue and bone thickness (Fig. 1c). The blurred images were subtracted from the original image (Fig. 1d). By adding 128 Gy values for each pixel to the images, areas of different brightness were created to help distinguish bone marrow from trabecular structure (Fig. 1e). The image was converted to a black-and-white format using the “Make Binary” option to make the boundaries of the bone marrow and trabecular structure distinguishable (Fig. 1f). The “Erode” option (Fig. 1g) was used to reduce the noise in the resulting image, and the “Dilate” option was used to enlarge the existing areas and make them more distinct (Fig. 1h). By applying the “Invert” step, the white areas in the image were converted to black, and the black areas were converted to white (Fig. 1i). Thus, the borders of the trabecular bone were revealed at this stage. Then, using the “Skeletonize” option, the image with the trabecular structure was converted to the skeletal structure format and made suitable for FA (Fig. 1j). To calculate the FD value, the image was divided into squares of 2, 3, 4, 6, 8, 12, 16, 32, and 64 pixels using the “Fractal Box Counter” option. The number of squares containing trabeculae and the total number of squares in the image were calculated for different pixel sizes. The slope of the line that best fits the points in the graph created by plotting these values on a logarithmic scale gave the FD value. FD measurements were taken bilaterally (right and left mandible) in the trabecular bone region above the mandibular canal.

### Mandibular radiomorphometric measurements

**Fig. 2:**
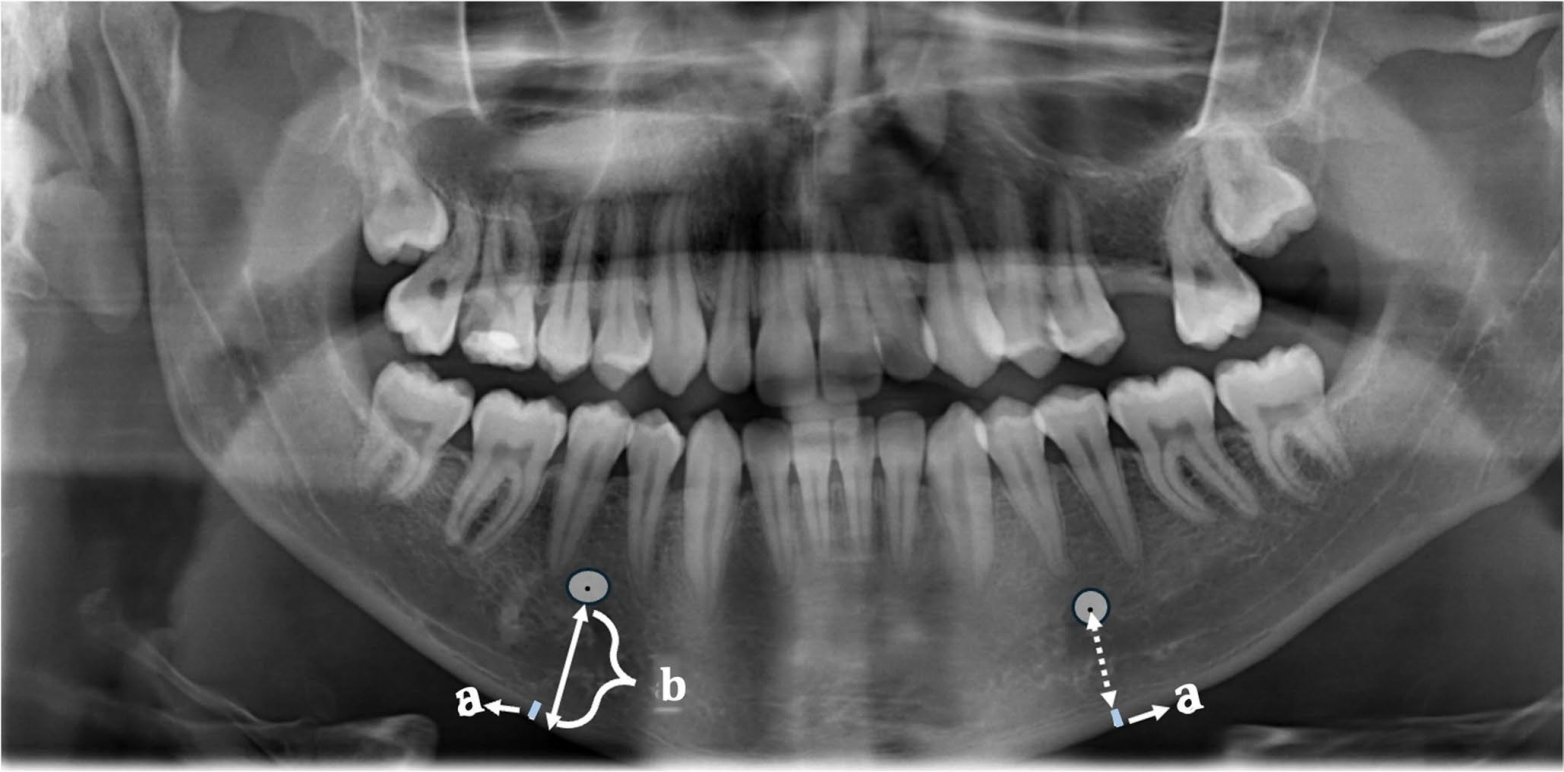
Measurement of PMI in the right mandible (PMI: **a**/**b**); measurements of MI in the left mandible (mandibular cortical thickness-MI: **a**)

Mental index (MI; thickness of the mandibular cortex): Mandibular cortical thickness on a perpendicular line drawn from the center of the mental foramen to the lower border of the mandible (a) (Fig. 2).

Panoramic mandibular index (PMI): The ratio of the mandibular cortical thickness on a perpendicular line drawn from the center of the mental foramen to the lower border of the mandible to the distance between the lower border of the mental foramen and the lower border of the mandible on the same line (b) (a/b) (Fig. 2). MI and PMI measurements were also performed bilaterally.

All measurements were evaluated by one oral radiologist with 5 years of experience, and data were recorded by the same researchers to ensure consistency and reliability. Prior to linear measurements and FD analysis performed on panoramic radiography, the researcher was blinded to the patients’ body mass index values. To assess intra-observer agreement, all measurements were repeated one month later by randomly selecting 20% of the panoramic images used in the study.

### Statistical analyses

The data obtained were analyzed using the IBM SPSS program version 23.0 (SPSS Inc., Chicago, USA). Compliance with normal distribution was examined with Shapiro-Wilk and Kolmogorov-Smirnov Tests. Pearson Chi-Square Test was used in the examination of categorical data and multiple comparisons were made with Bonferroni Corrected Z Test. Paired Two Sample t Test was used in the comparison of right and left measurements of parameters conforming to normal distribution, and Wilcoxon Test was used in the comparison of those not conforming to normal distribution. Independent Samples t Test was used in the comparison of parameters conforming to normal distribution in paired groups, and the Mann-Whitney U Test was used in the comparison of parameters not conforming to normal distribution. One-way analysis of Variance was used in the comparison of parameters conforming to normal distribution in three or more groups. Kruskal-Wallis Test was used in the comparison of parameters not conforming to normal distribution, and multiple comparisons were made with the Dunn Test. Intraclass Correlation Coefficient (ICC) was used in the examination of the agreement between measurements. Analysis results were presented as frequency (percentage) for categorical variables, and as mean ± standard deviation and median (minimum-maximum) for quantitative variables. The significance level was taken as *p* < 0.05.

## Results

The study included a total of 127 patients (84 female and 43 males), aged between 18 and 45 years, with a mean age of 30.37 ± 8.4 years. The comparison of sociodemographic characteristics according to BMI categories is presented in Table 1. Statistical analysis revealed a significant difference in median age across BMI categories (*p* < 0.001). A statistically significant association was found between gender and BMI categories (*p* = 0.029). In contrast, no significant association was found between smoking status and BMI categories (*p* = 0.816). As no statistical differences were detected between the right and left measurements for FD values, MI, and PMI (*p* > 0.05), the average of both sides was used in the analyses (Table 2).

**Table 1:** Comparison of sociodemographic data according to BMI categories

	**BMI**					
	**Underweight** (*n*=31)	Normal (*n *= 37)	Overweight (*n *= 30)	**Obese** (*n*=29)	**Total**	*p*-value
Gender						
Female	27 (32.1%)	22 (26.2%)	16 (19%)	19 (22.6%)	84 (66.1%)	0.029*
Male	4 (9.3%)	15 (34.9%)	14 (32.6%)	10 (23.3%)	43 (33.9%)	
Smoking						
Yes	7 (22.6%)	11 (35.5%)	6 (19.4%)	7 (22.6%)	31 (24.4%)	0.816*
No	24 (25%)	26 (27.1%)	24 (25%)	22 (22.9%)	96 (75.6%)	
Age	25.54 ± 6.16	28.38 ± 6.7	35.42 ± 8.23	32.87 ± 9.15	30.37 ± 8.4	< 0.001**

Pearson Chi-Square Test; **Kruskal Wallis Test

**Table 2:** Comparison of right and left measurements of parameters

	Right		**Left**		*p*-values
	Mean ± SD	**Median (min-max)**	**Mean ± SD**	**Median (min-max)**	
MI	5.12 ± 1.01	5 (2.85 – 8.5)	5.03 ± 0.98	5 (3 – 8.26)	0.264*
PMI	0.39 ± 0.36	0.35 (0.17 – 4.27)	0.36 ± 0.08	0.35 (0.18 – 0.63)	0.702*
FD angulus region	1.34 ± 0.09	1.35 (1.05 – 1.51)	1.33 ± 0.09	1.34 (1.09 – 1.5)	0.846*
FD condyle region	1.32 ± 0.08	1.33 (1.07 – 1.48)	1.34 ± 0.1	1.34 (1.07 – 1.67)	0.208**
FD premolar-molar	1.33 ± 0.09	1.34 (1 – 1.51)	1.34 ± 0.08	1.35 (1.03 – 1.5)	0.190*
FD premolar-canine	1.33 ± 0.08	1.33 (1.05 – 1.49)	1.33 ± 0.08	1.34 (1.08 – 1.48)	0.599*

MI: Mental index; PMI: Panoramic mandibular index; FD: Fractal dimension; SD: Standard deviation *Wilcoxon Test; **Paired Two Sample t-Test

Table 3 presents the MI and PMI indices, as well as FD analysis results, categorized by BMI. A significant difference was found between the values ​​of the MI and BMI categories (*p* = 0.005). Post-hoc analyses showed that the “underweight” and “normal” groups had similar MI values, but these values ​​were significantly different from those of the “overweight” and “obese” groups. Similarly, PMI values differed significantly across BMI categories (*p* = 0.025), with values for the normal group differing notably from the obese group. No statistically significant difference was found between FD premolar-molar, FD angulus, FD condyle values, and BMI groups (*p* > 0.05). However, a significant difference was found between FD premolar-canine values ​​according to BMI categories (*p* < 0.001). This difference appears to stem primarily from the separation of the “underweight” group from the “normal” and “overweight” groups.

**Table 3:** Comparison of radiomorphometric indexes and FD values according to BMI categories

	Underweight (*n*=31)	Normal weight (*n*=37)	Overweight (*n*=30)	Obese (*n*=29)	Total	*p*-values
MI	4.8 ± 0.77	4.92 ± 0,85	5.07 ± 0.91	5.57 ± 0,88	5.08 ± 0.89	0.005*
	4.82 (3.22 – 6.73)^b^	4.8 (3.45 – 6.63)^b^	4.85 (3,75 – 7.4)^a^	5.2 (4.5 - 8)^a^	5 (3.22 - 8)	
PMI	0.36 ± 0,07	0.38 ± 0.34	0.35 ± 0.07	0.39 ± 0.06	0.37 ± 0.19	0.025*
	0.35 (0.21 – 0.5)^ab^	0.33 (0.2 – 2.37)^b^	0.34 (0.22 – 0.51)^ab^	0.39 (0.29 – 0.55)^a^	0.35 (0.2 – 2.37)	
FD angulus region	1.32 ± 0,07	1.33 ± 0.09	1.35 ± 0.07	1.34 ± 0.05	1.34 ± 0.07	0.399**
	1.34 (1.12 – 1.42)	1.36 (1.15 – 1.48)	1.35 (1.19 – 1.51)	1.34 (1.26 – 1.48)	1.34 (1.12 – 1.51)	
FD condyle region	1.31 ± 0.07	1.33 ± 0.08	1.35 ± 0.06	1.34 ± 0.06	1.33 ± 0.07	0.115*
	1.3 (1.18 – 1.44)	1.35 (1.15 – 1.46)	1.33 (1.27 – 1.47)	1.34 (1.17 – 1.44)	1.34 (1.15 – 1.47)	
FD premolar-molar	1.31 ± 0.08	1.35 ± 0.04	1.34 ± 0.08	1.34 ± 0.06	1.34 ± 0.07	0.257*
	1.34 (1.15 – 1.43)	1.35 (1.27 - 1,46)	1.34 (1.13 – 1.46)	1.35 (1.22 – 1.47)	1.35 (1.13 – 1.47)	
FD premolar-canine	1.29 ± 0.06	1.34 ± 0.05	1.36 ± 0.06	1.33 ± 0.07	1.33 ± 0.06	<0.001*
	1.29 (1.18 – 1.42)^b^	1.35 (1.23 – 1.42)^a^	1.37 (1.22 – 1.44)^a^	1.34 (1.18 – 1.42)^ab^	1.35 (1.18 – 1.44)	

MI: Mental index; PMI: Panoramic mandibular index; FD: Fractal dimension

*Kruskal Wallis Test; **One-Way Analysis of Variance; Mean ± standard deviation; Median (minimum – maximum); a-b: There is no difference between groups with the same letter

When comparing gender groups (Table 4), the MI value was significantly lower in females than in males (*p* = 0.029). A significant difference was also found between genders in PMI values (*p* = 0.011). In addition, a significant difference was observed in FD condyle values, which were lower in females than in males ​​(*p* = 0.007). No significant differences were found between genders for FD angulus region, FD premolar-molar region, and FD canine-premolar region values ​​(*p* > 0.05).

**Table 4:** Comparison of parameters by gender

	**Gender**		Total	*p*-values
	**Female**	Male		
MI	4.93 ± 0.76	5.36 ± 1.06	5.08 ± 0.89	0.029*
	4.92 (3.22 – 7.21)	5.25 (3.75 - 8)	5 (3.22 - 8)	
PMI	0.39 ± 0.23	0.33 ± 0.08	0.37 ± 0.19	0.011*
	0.37 (0.2 – 2.37)	0.33 (0.2 – 0.49)	0.35 (0.2 – 2.7)	
FD angulus region	1.33 ± 0.07	1.35 ± 0.07	1.34 ± 0.07	0.076**
	1.34 (1.12 – 1.49)	1.36 (1.15 – 1.51)	1.34 (1.12 – 1.51)	
FD condyle region	1.32 ± 0.07	1.35 ± 0.07	1.33 ± 0.07	0.007*
	1.32 (1.15 – 1.46)	1.36 (1.15 – 1.47)	1.34 (1.15 – 1.47)	
FD premolar-molar	1.33 ± 0.07	1.35 ± 0.06	1.34 ± 0,07	0.156*
	1.35 (1.13 – 1.43)	1.36 (1,21 – 1.47)	1.35 (1.13 – 1.47)	
FD premolar-canine	1.32 ± 0.07	1.35 ± 0.05	1.33 ± 0.06	0.065*
	1.34 (1.18 – 1.44)	1.35 (1.19 – 1.42)	1.35 (1.18 – 1.44)	

MI: Mental index; PMI: Panoramic mandibular index; FD: Fractal dimension

*Mann Whitney U Test; **Independent Samples t Test; Mean ± standard deviation; Median (minimum – maximum)

The results of the intra-observer agreement analysis, conducted to evaluate the repeatability of the measurements, are given in Table 5. ICC values ​​for all parameters ranged from 0.730 to 0.943. These results indicate that, according to accepted criteria (ICC 0.75–0.90: good; ICC > 0.90: excellent), all measurements exhibit good to excellent reliability.

**Table 5:** Examining intra-observer agreement

	ICC (%95 CI)	*P*
MI	0.862 (0.693 – 0.938)	**<0.001**
PMI	0.943 (0.874 – 0.975)	**<0.001**
FD angulus region	0.876 (0.723 – 0.944)	**<0.001**
FD condyle region	0.818 (0.593 – 0.918)	**<0.001**
FD premolar-molar	0.730 (0.397 – 0.879)	**0.001**
FD premolar-canine	0.762 (0.469 – 0.893)	**<0.001**

MI: Mental index; PMI: Panoramic mandibular index; FD: Fractal dimension

Intraclass Correlation Coefficient (95% Confidence Interval)

## Discussion

In dental clinical practice, mandibular bone structure and quality are important determinants of implant success, periodontal health, and orthodontic treatment planning [24]. Therefore, when evaluating the structure of the jawbones, determining the effect of different BMI groups ​​on the microarchitectural structure of the bone is clinically critical. Osteopenia and osteoporosis are the most common causes of decreased bone mineral density [30]. Although Dual-energy X-ray absorptiometry (DEXA) is considered the gold standard in BMD assessment, its disadvantages, such as cost, radiation dose, complexity, and difficulty in accessibility, limit its use [28, 31]. On the other hand, panoramic radiographs offer an alternative assessment option with their low radiation dose, wide anatomical coverage, and ease of use in routine dental examinations [24]. In the literature, the effects of various systemic diseases and clinical conditions on mandibular cortical bone structure and trabecular bone density have been successfully evaluated using radiomorphometric indices and FD analysis methods on panoramic radiographs [15, 16, 28]. The aim of this study is to investigate the effect of BMI on mandibular bone quality using FD analysis and radiomorphometric measurements on panoramic radiographs.

FA is a non-invasive procedure that can detect early changes in alveolar bone by providing information about trabecular bone microarchitecture [20, 24]. This process is performed using a computer algorithm, unaffected by variables such as radiodensity and projection geometry [14]. FD values are frequently used in studies to investigate the bone quality of osteoporotic patients and some systemic disorders such as Diabetes [16, 24], familial Mediterranean fever [15], hyperlipidemia [14], chronic renal failure [32], and to define the effect of orthodontic treatments [23, 26], and periodontal disease on the surrounding bone [33–35]. In the literature, an increase in FD values has been associated with an increase in the structural complexity, while a decrease in FD indicates a flatter and less complex structure [15, 32]. Some studies evaluating bone mineral density in osteoporotic patients have observed a positive correlation with FD values [36, 37], while other studies have reported no difference between normal and osteoporotic groups [38, 39]. Furthermore, studies using FD analysis in different systemic disease groups or pathological conditions have reported different results regarding how changes in bone structure affect trabecular complexity. Özden and Çiçek [18] investigated the trabeculation differences in the mandibular bone architecture of patients with bilateral and unilateral cleft lip and palate using FA analysis. The study results showed that patients with cleft lip and palate obtained lower FD values ​​in the condyle and ramus regions compared to the control group [18]. In a study investigating the relationship between mandibular condyle FD and the severity of temporomandibular disorders and degenerative joint changes, it was reported that patients with degenerative changes exhibited significantly lower FD values [21]. Yıldızer et al. [24] evaluated the mandibular trabecular structure using FD in patients with type 1 and type 2 diabetes, as well as those with hypo- and hyperthyroidism, and found no significant difference in FD values between the diabetic groups. However, in their studies, they obtained significantly lower FD values ​​in hyperthyroidism groups compared to controls, especially in the ROI area, which they defined as the geometric center of the area between the mandibular notch and the mandibular foramen [24]. In a study by Günaçar et al. [14], which evaluated the mandibular bone structure of patients with hyperlipidemia using FD analysis, it was reported that the FD values of hyperlipidemic patients were lower than those of the healthy group, and a significant difference was observed, especially in terms of mandibular angle FD values. Temur et al. [28] used FA to evaluate potential changes in the mandibular bone in obese and non-obese children and adolescents. Their study evaluated two ROI areas (angulus and ramus) and reported no statistically significant difference between the obese and non-obese groups [28]. To our knowledge, this study is the first to investigate FD values ​​to determine mandibular bone quality in different body mass index groups of healthy adults. In our study, when FD values ​​in four mandibular regions were examined, FD values of underweight individuals were lower than those of other groups, and a significant difference was observed only in premolar-canine FD values. Differences in FD analysis results may be influenced by various characteristics of the study groups, including the selection of ROI from different sizes and regions, imaging techniques, and evaluation parameters, as well as the age and gender distribution in the studies [32].

The results obtained show lower FD values ​​in underweight groups, indicating a simpler trabecular structure, characterized by a decrease in the number or thickness of bone trabeculae, which may suggest decreased bone quality in the mandibular region. Moreover, in underweight individuals, the inadequate intake or impaired absorption of nutrients -specifically calcium, vitamin D, and protein- is known to compromise bone remodeling processes, which can negatively affect the trabecular structure and overall bone [40]. Low body weight is often associated with diminished levels of key hormones, such as estrogen and leptin, both of which play a role in bone metabolism [41]. However, the FD analysis findings suggest that when considering the effect of body weight on trabecular bone structure, the change in microarchitecture may occur in a more localized area rather than affecting the entire structure.

Radiomorphometric indices, such as the MI and PMI, have been widely used in studies to assess mandibular morphological changes, to determine osteoporosis or osteopenia on panoramic radiographs [10, 14–16]. These radiomorphometric indices, obtained from panoramic radiographs typically used in daily dental practice, can predict osteoporosis without the need for additional testing [42]. PMI, because it uses the ratio of two linear measurements, ensures that magnification differences between different panoramic devices do not affect the results, allowing for comparison between images [43, 44]. In our study, we used PMI and MI to assess changes in mandibular cortical bone in healthy adults with different BMIs. Many studies previously published in the literature have reported that PMI and MI measurements show excellent reliability and reproducibility [10, 28, 42, 45–47]. The results of our study yielded excellent intraclass correlation coefficients for both PMI and MI values.

The primary aim of this study was to test the null hypothesis that BMI does not significantly affect mandibular bone structure. However, our findings revealed significant differences between BMI groups and MI and PMI values ​​(*p* < 0.05). The results of our study showed that MI values (thickness of the mandibular cortex) were higher in obese and overweight patients compared to those who were normal weight or underweight. The PMI value of obese individuals was higher than that of individuals in the normal category, with statistical significance. Consequently, we rejected the null hypothesis stating that there is no relationship between BMI and mandibular bone structure.

It is reported that obesity increases insulin, estrogen, leptin, and inflammatory interactions and may affect systemic metabolism in bones, leading to an increase in bone mineral density [10]. Dense trabeculation in the mandibular bone has been reported to be associated with higher bone mineral density in the skeleton, and high levels of obesity generally increase bone mineral density [3]. Our study found that obese and overweight individuals have higher mandibular cortical thickness compared to individuals of normal weight, a result consistent with the study of Yasa et al. [10]. However, in the study by Temur et al. [28], PMI measurements were determined to be lower in obese children and adolescents compared to the healthy group, which is contrary to the findings of our study. This inconsistency in the studies may stem from differences in the study populations. While Yasa et al. [10] conducted their studies on adolescent individuals, Temur et al.‘s [28] study population consisted of children and adolescents aged 6 to 14 years. Our study population consisted of adult individuals aged 18–45 years. Unlike adults, children and adolescents are in a period of active growth, and changes in the hormonal environment and inflammatory processes during growth and development can differentiate the skeletal changes observed in childhood obesity from those in adults.

The mandibular cortical and trabecular structures significantly contribute to primary implant stability and successful osseointegration [48]. The higher MI and PMI values ​​observed in obese individuals in our study may indicate a more favorable cortical bone environment for implant placement. Increased mandibular cortical thickness in overweight and obese individuals can affect orthodontic treatments. Von Bremen et al. highlighted that an increased BMI is a risk factor for longer treatment times and more oral health problems [49]. Saloom et al. [50] concluded that obese patients required less time to achieve orthodontic tooth alignment compared to normal-weight patients; however, this difference was not statistically significant. However, it has also been reported that orthodontic tooth movements are significantly higher in obese patients compared to those of normal weight [50]. Furthermore, decreased bone mineral density may contribute to accelerated bone loss caused by periodontitis [51].

The mandibular premolar region is emerging as an important diagnostic area, and visual determination of the trabecular bone pattern and density can aid in the detection of patients with osteoporosis [52]. In our study, MI and PMI index results, as well as FD analysis results, were evaluated, and a significant difference in mandibular bone structure was observed in obese individuals compared to other BMI groups, particularly in measurements of the premolar/mental regions. This also demonstrates that BMI can affect different bone structures differently, such as weight-bearing and non-weight-bearing areas, as well as trabecular and cortical areas [28]. Given the limited number of studies investigating the link between obesity and radiomorphometric indices and FD analysis, the findings of our study may offer valuable information and contribute to the advancement of the existing literature.

Previous cross-sectional studies investigating the relationship between BMI and mandibular bone quality have frequently evaluated individuals over 40–45 years of age or children and adolescents [10, 28, 53]. It is well-established that bone mineral density gradually declines with age in both genders, with a more accelerated loss observed in females during the postmenopausal period [54]. Furthermore, Ravn et al. [55] reported that underweight is a significant risk factor for low bone mass and accelerated bone loss in postmenopausal women. To eliminate the potential impact of menopause-related hormonal changes on bone quality, we specifically excluded individuals over 45 years of age from the study. When the results in the literature regarding the relationship between gender and FD are examined, there are studies reporting no correlation between gender and FD [12] as well as studies indicating that FD is higher in males than in females [56–59]. In our study, FD measurements were higher in males than in females in all gender groups, and significant differences were observed, especially in condylar FD values. The discrepancies in the literature regarding gender-related FD values may stem from differences in sample demographics, particularly the age distribution and hormonal status of the participants. Furthermore, the selection of the Region of Interest (ROI) plays a crucial role; for instance, the higher FD values observed in the condyle in our study could be attributed to the typically greater masticatory forces in males. Additionally, variations in imaging modalities and image processing algorithms (such as thresholding techniques) across different studies may also account for the inconsistent findings.

Our study also evaluated whether there was a statistically significant relationship between gender, smoking status, and BMI categories. While a significant relationship was found between gender groups, there was no relationship between BMI groups and smoking status. The significant difference between genders suggests that the non-homogeneous distribution in the study group (a larger number of female individuals) may have resulted from proportional differences, particularly in the underweight category.

This study had some limitations. Mandibular bone tissue was primarily assessed using radiomorphometric indices and FA. Prospective studies including various bone assessment methods such as DEXA and computed tomography are needed to better understand the potential effects of obesity on the jaws. Another limitation of the study is that it was conducted using panoramic radiographs, a two-dimensional imaging. The effect of BMI on mandibular bone mineral density can be investigated with three-dimensional imaging methods. One limitation of our study is the small sample size and the non-homogeneous distribution of the sample. Future studies with a more homogeneous gender distribution and larger sample sizes could increase the generalizability of these findings to a broader population.

## Conclusion

Our study findings indicate that increased MI and PMI values ​​in overweight and obese individuals may lead to changes in mandibular cortical structure due to higher body weight. In contrast, when FD analysis results were evaluated across BMI groups, significantly lower FD values ​​were only found in lean individuals in the premolar-canine region. These findings demonstrate that physiological differences related to BMI can affect mandibular bone structure, and that the potential effects of obesity on mandibular bone should be considered, especially in surgical procedures such as orthodontics, implant placement, and tooth extraction. Future research should include more comprehensive assessments with various advanced imaging techniques, such as high-resolution computed tomography, to more accurately evaluate mandibular bone architecture with larger sample sizes.

## Data Availability

No datasets were created or analyzed during the current study.
