# Correction to: Effects of body mass index on mandibular bone architecture: a retrospective fractal dimension and radiomorphometric analysis

**DOI:** 10.1007/s00784-026-06923-7

**Published:** 2026-05-22

**Authors:** Nebiha Gozde İspir, Gulsun Akay, Kahraman Gungor

**Affiliations:** Department of Oral and Dentomaxillofacial Radiology, Gazi University, Bişkek Street,1.Sk. No:4, Ankara, 06490 Turkey

**Correction to: Clin Oral Invest**

10.1007/s00784-026-06833-8

In the published paper, several sections, including the article title, figure captions, and tables were incorrectly published in Turkish.

The original article has been corrected.

